# Complete mitochondrial genome sequence of *Pseudecheneis Sulcata* in the Yarlung Zangbo River, Tibet

**DOI:** 10.1080/23802359.2019.1623727

**Published:** 2019-07-10

**Authors:** Qingzhi Ma, Lei Li, Yu Du, Hongyu Jin, Bo Ma

**Affiliations:** a Heilongjiang River Fisheries Research Institute, Chinese Academy of Fishery Sciences, Harbin, China;; b College of Life and Technology, Harbin Normal University, Harbin, China;; c College of Fisheries and Life Science, Shanghai Ocean University, Shanghai, China

**Keywords:** *Pseudecheneis sulcata*, mitochondrial DNA, complete genome

## Abstract

*Pseudecheneis sulcata* belongs to Sisoridae, Pseudecheneis, which is mainly distributed in India and Tibet of China, and is located in the Motuo and Chayu in the lower reaches of the Yarlung Zangbo River in Tibet. In the present study, we obtained the complete mitochondrial genome sequence of *Pseudecheneis sulcata*, which was 16,535 bp in length. This genome consisted of 13 protein-coding genes, 22 tRNAgenes, 2 rRNA genes and a non-coding control region. The protein-coding genes have three start codons (GTG, ATG, and CTA) and four stop codons, including three complete stop codons and one incomplete stop codon. To verify the accuracy and utility of newly determined mitogenome sequences by constructing a species phylogenetic relationship tree of species, we expect to use the full mitochondrial gene sequence to interpret related evolutionary events.

*Pseudecheneis sulcata* belongs to Sisoridae, Pseudecheneis, which is mainly distributed in India and Tibet of China, and is located in the Motuo and Chayu in the lower reaches of the Yarlung Zangbo River in Tibet. In this study, *P. sulcata* was collected from the Motuo in the lower reaches of the Yarlung Zangbo River (sampling location geospatial coordinates is 29.18°N, 94.16°E). We published the compelte mitochondrial genome of *P. sulcata*. Extraction of total genomic DNA using phenol-chloroform method (Taggart et al. 1992). Also, the experimental specimens were kept at the Heilongjiang River Fisheries Research Institute, Chinese Academy of Fishery Sciences.

The complete mitochondrial genone of *Pseudecheneis sulcata* was 16,535 bp and had been provided in GeneBank (accession no. MK843301). The gene composition of all genomic sequences was the same as that of most vertebrates, including 13 protein-coding genes, 2 rRNA genes, 22 tRNA and a putative control region. The protein-coding genes have three start codons (GTG, ATG, and CTA), except that the COX1 and NAD6 genes use GTG and CTA as the start codons, respectively, and all other protein-coding genes use ATG as start codon. There are three stop codons (TAA, TAG and CAT)and incomplete stop codon (T--) in the protein coding genes.TAG is the stop codon of NAD2 and NAD3 genes.. TAA is the stop codon of NAD1, COX1, ATP8, ATP6, NAD4L and NAD5 genes. CAT is the stop codon of NAD6 gene. COX2, COX3, NAD4 and Cyt *b* genes use incomplete T as a stop codon. The 22 tRNA genes were identified using the no-coding tRNAscan-SE1.21 (http://lowelab.ucsc.edu/tRNAscan-SE). The overall base composition of this mitogenome was 32.03% for A, 26.36% for T, 26.56% for C, 15.05% for G, respectively, with an obvious higher A + T content (58.39%), consistent with the characteristics of high AT, low G content bias in the mitochondrial genome of most vertebrates (Shackelton et al. 2006).

As shown in Figure 1, the phylogenetic tree (neighbor-jioning) of *Pseudecheneis sulcata* in this study was constructed by ClustalX 1.81 and Mega 7.0 software (Kumar et al. 2016), together with other 10 species of Sisoridae and 1 *Pseudecheneis sulcata* (accession no. JQ026259.1) that have reported mitogenome sequence. Through the analysis of phylogenetic tree results, it was found that *Pseudecheneis sulcata* were gathered together and had a closer relationship with the Glyptothorax (Tilak 1976). The results of BLAST comparison showed that the identity difference between the complete mitochondrial genome sequence of *P. sulcata* was 3.94%. In addition, it will further enrich the research date of the family Sisoridae species and learn more about it.

**Figure 1.**
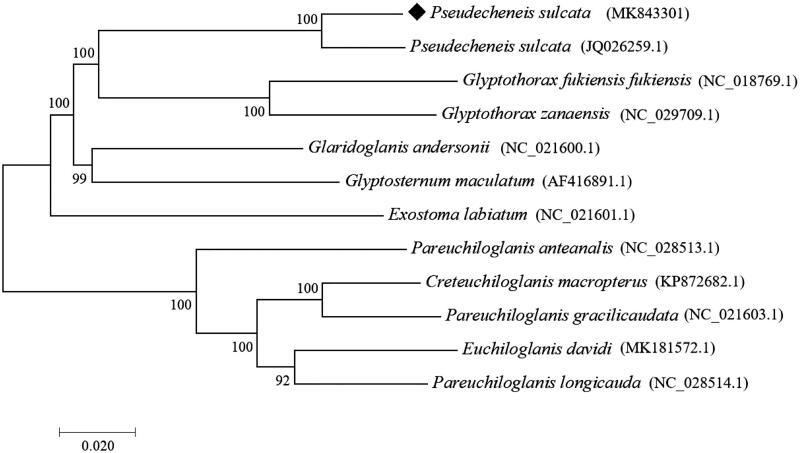
The phylogenetic tree (neighbor-jioning) of *Pseudecheneis sulcatus* in this study and other 11 species of Sisoridae which have reported mitogenome sequence. Shown next to the nodes are bootstrap support values based on 1000 replicates.The symbol befoe species names indicate newly determined mitochondrial genomes.

## References

[CIT0001] Kumar S, Stecher G, Tamura K 2016 MEGA7: molecular evolutionary genetics analysis version 7.0 for bigger datasets. Mol Biol Evol. 33:1870–1874. 27004904 10.1093/molbev/msw054 PMC8210823

[CIT0002] Shackelton LA, Parrish CR, Holmes EC 2006 Evolutionary basis of codon usage and nucleotide composition bias in vertebrate DNA viruses. Journal of Molecular Evolution. 62:551–563. 16557338 10.1007/s00239-005-0221-1

[CIT0003] Tilak R 1976 The adhesive thoracic apparatus in the evolution of glyptothoracoid fishes. Zool. Anz. 196:255–261.

[CIT0004] Taggart JB, Hynes RA, Prodoh PA, Ferguson A 1992 A simplified protocol for routine total DNA isolation from salmonid fishes. J Fish Biol. 40:963–965.

